# Hemorrhage Following Miscarriage, and Its Treatment by Intrauterine Injections

**Published:** 1876-06

**Authors:** W. W. Potter

**Affiliations:** Mt. Morris, N. Y.


ART. II.—*Hemorrhage Following Miscarriage, and its Treatment by Intra-uterine Styptic Injection, with Report of a Case.* Abstract of a paper read before the Livingston Co., Medical Society, by W. W. POTTER, M. D. of Mt. Morris, N. Y.

I was called in haste, Aug. 30th, 1873, at 7 o'clock P. M., to see Mrs. S——, aged 39, and who resided about two miles away. Knowing her to be between five and six months advanced in her 7th pregnancy, (having attended her two months previously when abortion was threatened) no time was lost in reaching her bedside, where she was found in the following condition to wit: Pulse 80, full and regular,—no pain, no hemorrhage.

Placing my hand over the abdomen, it was discovered to have diminished in size, and then she stated in explanation that she had felt pains irregularly for a day or two, and that in the afternoon of the day in question, they began to assume the regularity of labor-pains, and had so continued up to an hour or so prior to my visit. A digital examination revealed a fœtus lying in the vagina which I immediately removed.

The uterus being completely inert the attempt was made to restore its contractile power, by the use of cold, grasping, ergot, etc. Up to this time there had been no hemorrhage, but, not wishing to leave the patient with the placenta undelivered, I endeavored to reach it with the hand but was unable to do so. After repeated trials I determined to pack the vagina and await events.

The vagina was accordingly filled with small, soft sponges attached together by means of a string. I left the patient with directions to be summoned if hemorrhage should occur. At 10½ o'clock P. M., I was called in great haste and found the patient flowing to an alarming extent. I removed the packing from the vagina and cleared out the clots, but was still unable to reach the placenta. There had been no pain since my previous visit—in short complete inertia of the uterus. I re-tamponed as thoroughly as possible, which served to check the hemorrhage until 2 o'clock A. M., when it gushed forth again in all its horror. The patient was now placed in position, and my friend Dr. Joslyn, who had been summoned, administered ether and then grasped and held the uterus low down in the pelvis. I now succeeded in touching the lower border of the placenta with the fingers. Seizing the margin of the placenta within reach with a pair of uterine dressing forseps I made traction and brought away a small portion. Becoming satisfied that the placenta was adherent throughout a considerable portion of its surface, I repeated my efforts to detach it persistently, and at the end of an hour I had the satisfaction of bringing it away with the exception of some nodular shreds still clinging to the uterine walls. Hemorrhage had continued until the patient seemed on the verge of dissolution, and I submitted to my colleague the propriety of injecting the uterus with an iron styptic. He assented and I injected about one drachm of a sol. ferri persulphatis in water—1 part to 6.

The effect was magical—the mouths of the bleeding vessels were sealed, and the uterus, which had been inert for twelve hours, contracted firmly under the stimulus of the injection. The pain at once became severe, but from that moment the hemorrhage had ceased and it never returned. The uterine colic was relieved by morphia administered sub-dermically, and we left our patient at 5 o'clock A. M., in a condition of tolerable comfort. Convalescence was rapid, and complete restoration to health soon followed.

The patient again became pregnant in July of the present year, and in October she was thrown from her carriage and quite severely shocked. Abortion followed, but without any unusual attending conditions.

In view of the interest attaching to the treatment of post-partum hemorrhage by intra-uterine styptic injections, I have reported this case, believing that life was saved by the timely employment of this method. I have no doubt that the uterus contracted as a direct result of the injection, thus closing the mouths of the bleeding vessels, and from that moment the patient was safe.

